# Relationship between Acute-Phase Symptoms and Immunoglobulin G Seropositivity up to Eight Months after COVID-19

**DOI:** 10.3390/medicina58060708

**Published:** 2022-05-26

**Authors:** Ladislav Štěpánek, Magdaléna Janošíková, Marie Nakládalová, Lubomír Štěpánek, Antonín Tihelka, Alena Boriková, Renata Večeřová, Pavel Sauer

**Affiliations:** 1 Department of Occupational Medicine, University Hospital Olomouc, Faculty of Medicine and Dentistry, Palacký University Olomouc, I. P. Pavlova 185/6, 779 00 Olomouc, Czech Republic; magdalena.janosikova@fnol.cz (M.J.); marie.nakladalova@fnol.cz (M.N.); tihean00@lf.upol.cz (A.T.); alena.borikova@fnol.cz (A.B.); 2 Institute of Biophysics and Informatics, First Faculty of Medicine, Charles University, Salmovská 1, 120 00 Prague, Czech Republic; lubomir.stepanek@lf1.cuni.cz; 3 Department of Microbiology, University Hospital Olomouc, Faculty of Medicine and Dentistry, Palacký University Olomouc, Hněvotínská 3, 775 15 Olomouc, Czech Republic; renata.vecerova@fnol.cz (R.V.); pavel.sauer@fnol.cz (P.S.)

**Keywords:** COVID-19, seropositivity, symptom, dyspnea, anosmia

## Abstract

*Background and Objectives:* Given the limited knowledge of antibody responses to COVID-19 and their determinants, we analyzed the relationship between the occurrence of acute-phase symptoms and infection-induced immunoglobulin (Ig) G seropositivity up to 8 months post-symptom onset. *Materials and Methods:* In this cross-sectional study, 661 middle-aged unvaccinated healthcare workers (HCWs) were interviewed about the presence of symptoms during the acute phase of their previously confirmed COVID-19 and were tested for specific IgG, targeting the spike protein (S1 and S2). The dependence of seropositivity on the symptom occurrence was explored through multiple logistic regression, adjusted for the interval between symptom onset and serology testing, and through classification and regression trees. *Results:* A total of 551 (83.4%) HCWs showed seropositivity and, inversely, 110 (16.6%) HCWs were seronegative. The chance of IgG seropositivity was increased by dyspnea (odds ratio (OR) 1.48, *p* < 0.001) and anosmia (OR 1.52, *p* = 0.021). Fever in HCWs with dyspnea resulted in the highest detected seropositivity rate, and anosmia in HCWs without dyspnea significantly increased the proportion of seropositivity. *Conclusion:* Clinical manifestation of the acute phase of COVID-19 predisposes to the development of infection-induced antibody responses. The findings can be applied for assessing the long-term protection by IgG, and thus, for creating effective surveillance strategies.

## 1. Introduction

Severe acute respiratory syndrome coronavirus 2 (SARS-CoV-2) can affect the tissues of virtually all body systems, but most often the respiratory, gastrointestinal, nervous and cardiovascular, which results in a diverse clinical manifestation of the coronavirus disease 2019 (COVID-19) [1,2]. In the absence of definitive correlates of protective immunity, the presence of neutralizing antibodies to SARS-CoV-2 provides the best currently known indication of being protected against reinfection (in previously infected subjects) or breakthrough infection (in vaccinated subjects) [3]. The expression level and sustainability of anti-SARS-CoV-2 antibodies are highly variable among individuals depending on various factors such as age, severity and duration of the acute phase, nutritional status, medications, etc. [4].

The study, following a study by the same authors on the kinetics of antibody responses [5], aimed to analyze the relationship between symptoms of the acute phase of COVID-19 and infection-induced immunoglobulin (Ig) G seropositivity up to 8 months post-symptom onset.

## 2. Patients and Methods

### 2.1. Study Population

This study adopted a cross-sectional design. The study sample consisted of all healthcare workers (HCWs, *n* = 661) from the Olomouc Region, who were examined in a catchment occupational disease center between November 2020 and September 2021 to have their COVID-19 recognized as an occupational disease. In these HCWs, viral ribonucleic acid (RNA) was collected by a nasopharyngeal swab and detected using a reverse transcription polymerase chain reaction (RT-PCR) test previously in the acute phase of the disease. Included were only HCWs with the positive RT-PCR test 1 to 8 months before the examination in the occupational disease center. All included cases (previously SARS-CoV-2 naïve) were symptomatic and unvaccinated against COVID-19. HCWs with SARS-CoV-2 reinfection and those who reported suspected COVID-19 symptoms after their recovery were excluded.

The participants were examined according to a uniform protocol and submitted a blood sample for serology testing. During the examination, the HCWs were interviewed about the presence of common symptoms accompanying the acute phase of COVID-19 (listed by the World Health Organization and the Centers for Disease Control and Prevention [6,7]) and the date of symptom onset. The presence of each symptom was determined by a yes/no question. The studied HCWs brought a report about the course of their disease from their general practitioner (GP), against which the information provided was validated. In case of a discrepancy between the anamnestic data provided by the participant and the GP’s report, the data were repeatedly verified (through other medical reports if available) and the data from the participant were finally taken into account.

Epidemiological data showed that in the period in which HCWs became infected with SARS-CoV-2, the wild-type, alpha and delta variant of the virus dominated in the Czech Republic [8]. The study was approved by the Ethics Committee of the University Hospital Olomouc and Palacký University Olomouc (reference no. 18/21). All participants signed an informed consent form regarding the anonymous use of their data.

### 2.2. Laboratory Analysis

Specific antibodies were determined using SARS-CoV-2 chemiluminescence immunoassays by DiaSorin–Liaison SARS-CoV-2 S1/S2 IgG performed on the Liaison XL analyzer (DiaSorin S.p.A., Saluggia, Italy). The automated IgG assay detected antibodies against the S1 and S2 subunits of the spike protein. The level of IgG at ≥ 15 AU/mL represented seropositivity (according to the manufacturer’s instructions). For the diagnostic assay, DiaSorin guaranteed high sensitivity and specificity, as well as excellent detection of neutralizing antibodies (94.4% positive agreement with the plaque reduction neutralization test) [9]. Both antibody detection and previous RT-PCR testing were performed in a certified microbiological laboratory of University Hospital Olomouc in compliance with all standard procedures and manufacturers’ instructions of the used diagnostic kits and devices.

### 2.3. Statistical Analysis

Statistical analyses were conducted with the IBM SPSS Statistics, version 22 (SPSS Inc, Chicago, IL, USA). Except for descriptive statistics, multiple logistic regression was applied to explore the dependence of a serology status (seropositivity or seronegativity, inversely, as a disjunct event) on the presence of particular symptoms. The regression model was adjusted for individual intervals between symptom onset and serology testing. Significant associations of symptom clusters with the serology status were explored through classification and regression trees (CART) employing the chi-squared test. The method investigated possible symptom combinations maximizing the difference in the seropositivity rate. The level of statistical significance was set at *p* = 0.05.

## 3. Results

**Figure 1:**
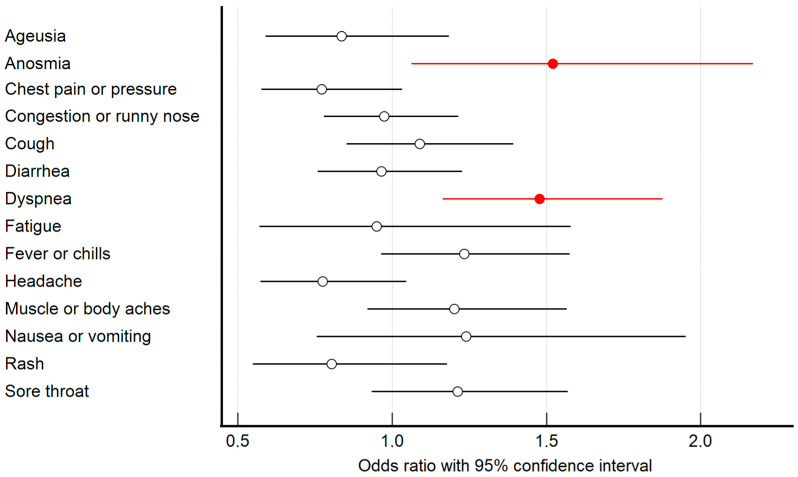
Size effect of each symptom on the seropositivity rate. Statistically significant effects highlighted in red.

**Figure 2:**
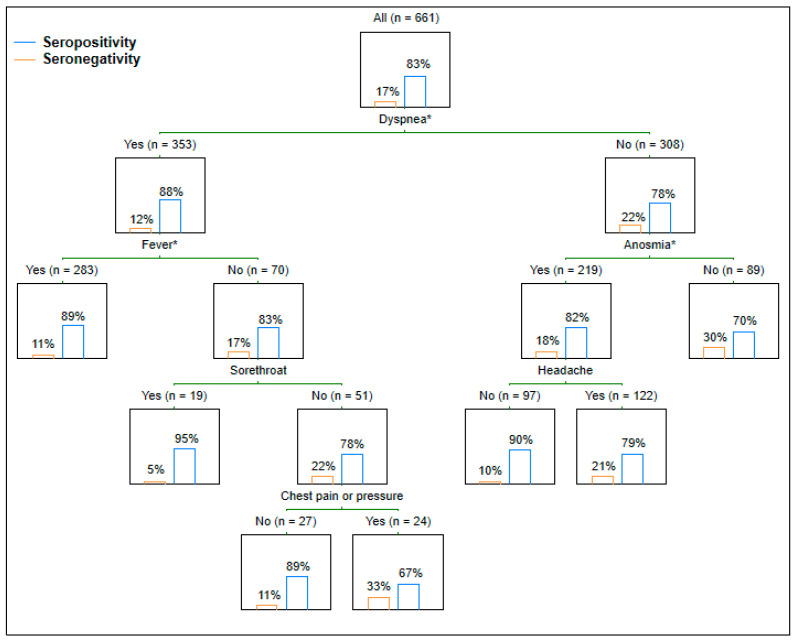
Immunoglobulin G seropositivity rate respectful to symptom clusters. * Statistically significant.

Of 661 HCWs with a mean age of 44.1 ± 10.8 years, 540 (81.7%) were female, and 121 (18.3%) were male. At the time of the examination, 551 (83.4%) HCWs showed seropositivity and, inversely, 110 (16.6%) HCWs were seronegative. The interval between symptom onset and serology testing averaged 83.1 ± 42.8 days. Both the rate comparison and the regression analysis revealed that the presence of dyspnea and anosmia were statistically significantly related to the serology status (Table 1, Figure 1). Dyspnea and anosmia in the acute phase of COVID-19 increased the chance of IgG seropositivity 1.48 and 1.52 times, respectively. The CART approach demonstrated that fever in the presence or anosmia in the absence of dyspnea played significant roles in the serology status (Figure 2). In other words, fever in HCWs with dyspnea resulted in the highest detected seropositivity rate, and anosmia in HCWs without dyspnea significantly increased the proportion of seropositivity. In addition to the symptoms interviewed, others were more rarely mentioned. 

In addition to the common symptoms of the acute phase of COVID-19, some others were stated. Specifically, 64 HCWs reported mental disorders (anxiety, irritability, insomnia) in the acute phase, 27 HCWs reported skin problems (itchy or burning skin, hair loss), 20 HCWs reported eye problems (painful, itchy, or burning eyes), and 16 HCWs reported painful or clogged paranasal sinuses.

## 4. Discussion

According to the obtained results, the presence of dyspnea in the acute phase of COVID-19 increased the probability of persisting IgG seropositivity in the following months. This is consistent with the findings of studies from the US (*n* = 250) and Indonesia (*n* = 83) conducted among COVID-19 convalescent plasma donors, in which a history of dyspnea was significantly related to protective anti-SARS-CoV-2 IgG titers [10,11]. The odds ratio (OR) of dyspnea for IgG seropositivity determined in the US study was 1.61, i.e., similar to the present work (Table 1) [10]. An Iranian seroprevalence study among 503 HCWs found that in addition to dyspnea (OR = 1.57), anosmia was also, and even in a stronger way than in our study, associated with seropositivity (OR = 2.81) [12]. Similarly in an Australian study conducted among 5345 blood donors, compared to those who had a confirmed SARS-CoV-2 infection but were seronegative, seropositive donors more frequently reported anosmia in acute COVID-19 (OR = 2.49) [13]. Other studies considered anosmia and ageusia to be a single symptom. Such studies also found a significant positive relationship between anosmia/ageusia and persisting IgG seropositivity [14,15]. In the present study, isolated ageusia was not a statistically significant predictor of seropositivity (Table 1, Figure 1). These neurotoxic effects of SARS-CoV-2 might be caused by changes in the phosphorylation pattern of proteins associated with axons and synapses in olfactory/gustatory neurons or injuries to any of VII, IX, X cranial nerves and the brain, including the cortex. The option of a SARS-CoV-2 trans-mucosal invasion from olfactory epithelium through the olfactory nerve into brain regions resulting also in olfactory projections, is highly debated [16,17,18]. However, the revealed different impact of anosmia and ageusia on antibody responses suggests that both disorders may involve distinct pathophysiological processes.

In available studies, other symptoms, most commonly fever, were also identified as predictors of anti-SARS-CoV-2 IgG [10,11,12]. Since almost all symptomatic subjects experience more than one symptom, combinations of symptoms need to be evaluated too. A British seroprevalence study among 956 HCWs detected that the combination of fever and/or cough and/or anosmia had a positive predictive value of 92.3% for seropositivity [19]. The present study identified two statistically significant symptom clusters (associated with dyspnea) (Figure 2).

Besides the presence of specific symptoms, other variables seem to be relevant to subsequent immune responses, including disease severity [20,21], a number of symptoms reported in the acute phase [5], its duration [22], and initial viral loads [23], etc. The development of immune responses begins with the infection of the mucous membranes. To enter cells, SARS-CoV-2 relies on its obligate receptor, angiotensin-converting enzyme 2 (ACE2), which is expressed in the epithelium of many tissues. Analysis of animal models and human transcriptome databases suggests that the ACE2 expression in the lower lung is relatively limited to type II alveolar cells, but is higher in the upper bronchial epithelia and much higher in the nasal epithelium, especially in the ciliated cells. This difference in ACE2 expression level in the respiratory tract is mirrored by the SARS-CoV-2 infection gradient, with nasal ciliated cells being primary targets for SARS-CoV-2 replication in the early stage of infection. Despite the respiratory route being dominant in a SARS-CoV-2 infection, the highest levels of ACE2 expression were found in the small intestine, testis, kidney, heart muscle, colon, and thyroid gland. Thus, many body organs and tissues may be directly infected with SARS-CoV-2, which, along with the activation of immune cells and release of several chemokines and cytokines, is reflected in the diverse clinical manifestation with organ-specific and -unspecific symptoms [24].

The association between viral load and COVID-19 outcomes is not entirely clear, as evidenced by Dadras et al. in their systematic review of 34 studies, the majority of which utilized RT-PCR of the nasopharyngeal/respiratory swabs to report the viral load. The results were inconclusive about the existence of a relationship between the infective viral load and COVID-19 severity, as a similar number of studies either approved or opposed the hypothesized relationship [25]. In a Spanish study (*n* = 132), high initial viral load predicted an earlier IgG response, while nonseroconversion was linked with very low initial SARS-CoV-2 RNA levels, suggesting that the induction of the adaptive humoral immune response might be dependent on the intensity of viral replication [23]. In the current study, SARS-CoV-2 was diagnosed by RT-PCR without determining the viral load. The findings in the available literature are inconsistent regarding the relationship between the viral load, detected from the nasopharyngeal swab, and infection-induced anosmia. An Indian study (*n* = 200) noted that patients with a recorded olfactory dysfunction at diagnosis had a significantly higher SARS-CoV-2 load, whereas Italian (*n* = 60) and Chinese (*n* = 143) studies did not correlate the viral load with both the presence and severity of anosmia [26,27,28].

Disease severity is assessed according to lower respiratory tract involvement [29]. Antibody responses to SARS-CoV-2 and their persistence were significantly correlated to disease severity [20,21]. This may contribute to the significant relation of dyspnea, which is a clinical manifestation of the lower respiratory tract involvement, to persisting IgG seropositivity, as shown by the present study.

Despite the development of the COVID-19 pandemic, the occurrence of the most common symptoms of the acute phase remains similar, although certain symptoms may appear more prominent depending on a virus strain, affected population, geographical area, etc. [1,30,31]. The study limitations include a cross-sectional design; however, the regression model was adjusted for the interval between symptom onset and serology testing. The fact that viral load was not detected in the subjects at diagnosis of the SARS-CoV-2 infection can also be considered a study limitation.

## 5. Conclusions

Both dyspnea and anosmia in the acute phase of COVID-19, reflecting respiratory and neurological damage, respectively, predispose to the development of a months-persisting antibody response in unvaccinated subjects. On the contrary, other symptoms such as gastrointestinal do not have a significant effect on the serology status. The findings can be applied for assessing long-term infection-induced immunity, and thus, for creating effective surveillance strategies.

## Figures and Tables

**Table 1:** Serology status with respect to the presence of particular symptoms.

Symptoms	Symptom Occurrence			Regression Analysis		
	Entire Sample	Serology Status		Odds Ratio of Seropositivity	95% Confidence Interval (Lower; Upper Value)	*p*-Value
		Seropositivity (N, %)	Seronegativity (N, %)			
Ageusia/dysgeusia	444 (67.2%)	376 (68.2%)	68 (61.8%)	0.836	0.591; 1.184	0.314
Anosmia/dysosmia	479 (72.5%)	409 (74.2%)	70 (63.6%)	1.520	1.064; 2.170	0.021
Chest pain or pressure	245 (37.1%)	202 (36.7%)	43 (39.1%)	0.772	0.577; 1.032	0.223
Congestion or runny nose	335 (50.7%)	281 (51%)	54 (49.1%)	0.974	0.78; 1.216	0.817
Cough	469 (71%)	400 (72.6%)	69 (62.7%)	1.090	0.853; 1.394	0.49
Diarrhea	202 (30.6%)	169 (30.7%)	33 (30%)	0.965	0.759; 1.228	0.773
Dyspnea	353 (53.4%)	310 (56.3%)	43 (39.1%)	1.478	1.164; 1.877	<0.001
Fatigue	631 (95.5%)	527 (95.6%)	104 (94.5%)	0.950	0.571; 1.579	0.842
Fever or chills	483 (73.1%)	412 (74.8%)	71 (64.5%)	1.233	0.965; 1.576	0.094
Headache	540 (81.7%)	448 (81.3%)	92 (83.6%)	0.775	0.574; 1.047	0.096
Muscle or body aches	526 (79.6%)	446 (80.9%)	80 (72.7%)	1.201	0.920; 1.567	0.178
Nausea or vomiting	54 (8.2%)	48 (8.7%)	6 (5.5%)	1.240	0.787; 1.952	0.354
Rash on skin	50 (7.6%)	40 (7.3%)	10 (9.1%)	0.804	0.549; 1.178	0.263
Sore throat	185 (28%)	161 (29.2%)	24 (21.8%)	1.212	0.935; 1.570	0.146

## Data Availability

The data that support the findings of this study are available from the corresponding author (Ladislav Stepanek) upon reasonable request.
